# Properties, Structure, and Acceptability of Innovative Legume-Based Biscuits with Alternative Sweeteners

**DOI:** 10.1155/2024/8216796

**Published:** 2024-03-25

**Authors:** Andriana E. Lazou

**Affiliations:** Laboratory of Chemistry, Analysis & Design of Food Processes, Department of Food Science and Technology, School of Food Sciences, University of West Attica, Athens, Greece

## Abstract

The effects of legume incorporation and sweetener substitution on the quality characteristics of innovative biscuits were investigated. The wheat flour was substituted with chickpea and lentil flour at ratios ranging from 0 to 30% legume to whole-meal dicoccum wheat flour. The sugar was substituted by oligofructose at 50 and 100% levels. The quality characteristics, including physicochemical properties (moisture content, water activity, and color), sorption characteristics, structural and textural properties, and sensory properties, were significantly affected by the substitutions. Sorption phenomena were excellently described by the Guggenheim, Anderson, and de Boer (GAB) model, while its parameters were affected by substitutions. Scanning electron microscopy revealed a porous structure with starch granules embedded within the protein matrix, showing restricted gelatinization and keeping largely their form. The incorporation of legume flour increased the biscuit density, hardness, and spread ratio and decreased the color of the products. Furthermore, principal component analysis (PCA) analysis of instrumental and sensory characteristics showed that texture and sweetness were the key quality characteristics for product acceptance. It was found that highly acceptable legume-based biscuits with alternative sweeteners can be produced, with 50% oligofructose substitution and legume flour incorporation (chickpea or lentil) up to 30%.

## 1. Introduction

Biscuits are a popular snack enjoyed worldwide due to the fact that they are products with a wide variety to meet the taste demands of consumers; they have a pleasant taste, a high nutritional value, and a long shelf life, and they are also products offering flexibility to the consumer since they are ready to eat and easy to store. The global sweet biscuit market had a value of $109 billion in 2023 and is expected to reach $192 billion by the end of 2033, showing a compound annual growth rate (CAGR) of 5.8% [1]. Within this market, the fortified biscuit market was valued at $1.15 billion in 2021 and is expected to grow at a CAGR of 5.4% [2]. In addition, the global healthy biscuit market was valued at $2.49 billion in 2021 and is estimated to reach a value of $3.82 billion by the end of 2030, expanding at a CAGR of 5.20% from 2022 to 2030 [3]. Also, biscuits are popular snacks enjoyed worldwide, but they are often considered energy-dense and lacking in some essential nutrients. The incorporation of legumes offers an opportunity to address these concerns while maintaining product quality and consumer satisfaction. Attempts have been and are being made to produce biscuits with high energy that also have nutritional benefits, such as high-protein and diabetic biscuits. This often adds extra cost, but by incorporating low-cost ingredients, such as legumes, one can compensate for the increased cost [4–8].

In the past, there have been several studies indicating reformulation of biscuits by adding different types of legumes, such as chickpeas, lupins, green lentils, and navy beans [9–13]. It should be noted that, compared to bread, gluten plays a minor role in biscuits, with its development generally being regarded as undesirable as it can create problems (excessive shrinkage or distortion, thickness reduction, and checking), which causes biscuits to break or crack after baking [14, 15]. However, the role of gluten quality and composition is critical to the characteristics of cookie dough [16]. Furthermore, it has been found that adding legume flour to biscuits boosts protein content correspondingly while decreasing dough spread. Its incorporation also results in a darker surface color and an increase in product hardness. Further, using legumes to enrich bakery products is especially promising for GF formulations, as the role of gluten is not as significant in biscuit production as in the case of other bakery products [11, 12, 17, 18].

However, the utilization of legumes in biscuit formulations is faced with some challenges that need further solutions and concern the presence of antinutritional factors, as well as challenges in terms of formulation, processing, and shelf life. In the case of biscuits, antinutritional factors can be eliminated through heating during baking [19]. However, there is a need for further research to explore the effects of adding legumes to biscuit formulations on their physicochemical and mechanical properties, including their effect on structure, texture, color, shelf life, sensory attributes, and acceptance as well. Among legumes, chickpeas and lentils are very promising in developing new, novel, and varied products belonging to the biscuit category. Past efforts showed that chickpea incorporation in high ratios led to a decrease in moisture content, a harder texture, and less pore formation, leading to unacceptable products [20]. Further, Sibian and Riar [21], based on functional and pasting properties, reported that chickpea-blended flour shows the suitability of substitution and utilization of different flours from germinated grains. Owing to the alteration in pasting properties and functional characteristics, chickpea-blended flour gave cookies a soft texture. In addition, Wang et al. [22] developed a high-protein biscuit made from oat milk by-product flour fortified with chickpeas. Furthermore, attempts were made to develop GF biscuits using composite rice and chickpea flour. To overcome handling difficulties due to the absence of gluten, xanthan gum was added to the rice-chickpea flour, which significantly affected the textural and linear viscoelastic properties of the dough as well as the texture, weight, moisture, *a*_w_, and dimensions of the biscuits. Saleem et al. [23] studied the addition of lentils to wheat flour for biscuit preparation. They observed significant increases in the protein, fat, crude fiber, and ash contents of the biscuits. Also, they found significant differences in the thickness and spread ratio of biscuits. They suggested substitution at levels of 21 and 28%, which were considered the most excellent for all sensory attributes, showing that lentil flour at various proportions might provide biscuits with acceptable sensory quality. More recently, Hajas et al. [24] investigated the preparation of gluten-free (GF) cookies based on green and red lentils using different combinations of whey protein, inulin as dietary fiber, and xylitol as a sweetener. Lentil products showed the same characteristics for textural attributes, noting that inulin addition increased the hardness of cookies. Further, all the xylitol-containing cookies were less crumbly than the controls, giving them crumblier, less hard, and crunchier products. The findings were considered a starting point for future research and development of functional GF products. Oligofructose, a natural sugar comprising mainly of *β*-(2→1) fructosyl units (*n* = 2 − 10), could be used for partial and/or total sugar substitution in biscuits, contributing to reduced caloric value while imparting sweetness [25, 26]. Depending on its commercial grade, it could affect various quality characteristics as well as physicochemical, structural, texture, and sensory properties, necessitating further investigation when added to biscuit formulations.

Having in mind that *Triticum dicoccum* wheat and its products are getting popularity among consumers as being among the ancient wheat species having health benefits and its organic farming, as well as previous studies on legume substitution in biscuits giving controversial results for some of their properties, the present work was undertaken to investigate a novel biscuit product using *Triticum dicoccum* whole-meal flour partially substituted by legume flour from lentils and chickpeas at various percentages (0–30%) as well as sugar substitution by oligofructose at percentages of 0, 50, and 100%. Accordingly, the effect of legume and oligofructose addition on quality characteristics, including the structural properties (apparent density, true density, porosity, and spread ratio), the physicochemical properties (water activity, moisture content, and color (*L*∗, *a*∗, and *b*∗)), the texture, the sensory characteristics, and moisture sorption isotherms at different temperatures, was investigated. The microstructure was evaluated using scanning electron microscopy. The sorption isotherms were modeled using the GAB model, and the properties were correlated using principal component analysis to reveal differentiations among innovative products with desired quality characteristics.

## 2. Materials and Methods

### 2.1. Raw Materials

Triticum dicoccum whole-meal flour was donated by Jofran-Frantzeskakis S.A. (Athens, Greece), while chickpea and lentil flour was provided by Pantazis Stone Mills (Megara, Attica, Greece). Fibrulose® (oligofructose) was purchased from Astron Chemicals S.A. (imported from Consucra-Groupe Warcoing S.A.). Shortening, sugar, baking powder, and sodium bicarbonate were purchased at a local market in Athens, Greece.

### 2.2. Biscuit Production

Whole dicoccum wheat flour as well as legume flour were weighed, mixed, and sieved. Chickpea and lentil flours were mixed with dicoccum wheat flour in order to obtain different legume-to-flour proportions, namely, 0%, 10%, and 30%. The sugar content of biscuits was substituted with oligofructose in proportions of 0, 50%, and 100% (sugar, sugar-oligofructose, and oligofructose, respectively). The 100% sugar substitution was made for the control recipe (without legume flour). The substitutions are summarized in Table 1(a).

**Figure 1:**
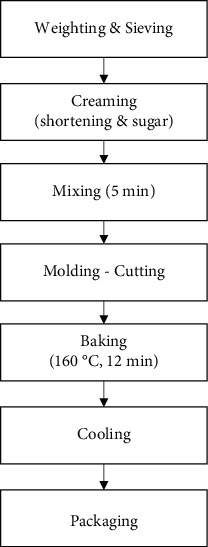
Production flow chart of innovative legume-based biscuits with alternative sweeteners.

The production procedure includes cream formation with sugar (or an oligofructose mixture), shortening, and then the addition of the rest of the ingredients. The mixing process duration was 5 min, in order to ensure the hydration and homogenization of the ingredients. Then, the dough was left to rest for 5 min, rolled and laminated at 2 mm, and cut with a 6 cm circular molding cutter. The production procedure is summarized in Figure 1. The experimental preparation was conducted in batches and included three replicates. Biscuits were baked at 160°C for 12 min, cooled to room temperature for 1 h, packed in laminate OPP 20/MET OPP 20, and kept at room temperature until further property determination.

### 2.3. Determination of Biscuit Quality Characteristics

#### 2.3.1. Moisture Content and Water Activity

Moisture content was performed using the AACC 44-15.02 gravimetric method [27], in triplicate using about 2 g accurately weighed biscuit samples, and drying within weighing bottles at 110°C until a constant weight was reached in an air oven (Gallenkamp Oven BS Model OV-160, United Kingdom). Three replicate samples were utilized for the measurement of water activity (*a*_w_) of the biscuits using an Aqua Lab 4TE aw-meter (Decagon Devices, Inc., USA) [28].

#### 2.3.2. Sorption Isotherms

The standard gravimetric method was used for the determination of sorption isotherms in triplicate at three different temperatures of 25, 35, and 45°C, as previously described by Lazou and Krokida [29] and Lazou et al. [28]. Briefly, the samples were milled until they became a homogeneous powder. The biscuit powder was kept for 3 weeks in phosphorus pentoxide desiccators and then placed in desiccators with saturated solutions of different salts, creating water activities (*a*_w_) from 0.11 to 0.94 for 3 weeks until the samples obtained a constant weight. At the end of this period, moisture determination was performed by placing the samples in a vacuum oven at 71°C for 24 hours. The data were fitted using the GAB model:

(1) $$\begin{array}{c} X=\frac{X_{\text{m}}{Cka}_{\text{w}}}{\left(1-{ka}_{\text{w}}\right)\left(1-{ka}_{\text{w}}+{Cka}_{\text{w}}\right)}, \end{array}$$

where *X* is the predicted equilibrium moisture content (% d.b.), *a*_w_ is the water activity, *X*_m_ is the monolayer moisture content (% d.b.), and *C* and *k* are constants related to the temperature effect. The GAB model parameters were estimated using nonlinear regression.

#### 2.3.3. Structural Properties

The diameter and thickness of biscuits were determined by measuring the diameter (*d*) and thickness (*h*) of seven biscuit samples placed edge to edge with a digital vernier caliper. The average values for diameter and thickness were reported in centimeters. The spread ratio was calculated by dividing diameter by thickness, using the following equation:

(2) $$\begin{array}{c} \text{SR}=\frac{d}{h}. \end{array}$$

The apparent density was determined according to the method of Lazou and Krokida [29] by measuring the actual dimensions of seven biscuits. The dimensions of the biscuits were measured with a Vernier caliper, considering them as cylinders. The apparent density was determined by the following equation:

(3) $$\begin{array}{c} \rho_{\text{a}}=\frac{4m}{\pi d^{2}h}, \end{array}$$

where *m* is the mass of the samples (g), *d* is the diameter (cm), and *h* is the thickness (length) (cm) of the samples. Density values are the average of five replicated measurements.

The true density of the biscuit samples was calculated from the following equation:

(4) $$\begin{array}{c} \rho_{\text{t}}=\frac{m}{V_{\text{S}}}, \end{array}$$

where *m* is the mass of the sample and *V*_S_ is the true volume (cm^3^). The true volume was measured using a Quantachrome stereopycnometer (model SPV-3) with an accuracy of 0.001 cm^3^, assuming no closed pores remain in the sample for helium. Biscuits were milled, weighed, and placed on a microcell scale. The volume density was determined using the following equation:

(5) $$\begin{array}{c} V_{\text{S}}=V_{\text{C}}+V_{\text{R}}\left(\frac{P_{1}}{P_{2}}-1\right). \end{array}$$

The volume *V*_C_, the volume *V*_R_, and the pressures *P*_1_ and *P*_2_ arise from the calibration of the instrument using a metal sphere with a specific diameter for the microcell. The results are the average of five replicate measurements.

The porosity of the biscuit samples was calculated using the following equation:

(6) $$\begin{array}{c} \varepsilon =1-\frac{\rho_{\text{a}}}{\rho_{\text{t}}}, \end{array}$$

where *ρ*_a_ is the apparent density and *ρ*_t_ is the true density of the samples. The results are the average of five replicated measurements.

#### 2.3.4. Textural Properties

The textural properties of the biscuits and, more specifically, the breaking strength were determined through the three-point bend test using a texture analyzer (TA.XT2i; Stable Micro Systems, UK). The probe for the conduct of the test was the HDP/3 PB, and the load was 5 kg. The span between supports was 40 mm. The samples were compressed until they reached 50% of their height with a pretest speed of 1.0 mm/s, a speed test of 3.0 mm/s, and a posttest speed of 10 mm/s. The breaking strength was determined from the received graph as the maximum force before the biscuit breakage. The results are the average of seven replicated measurements.

#### 2.3.5. Color

The color determination of the biscuits was performed on three replicate samples using a HunterLab colorimeter (Miniscan XE Plus, Virginia, USA), and the values of the color parameters *L*∗ (lightness), *a*∗ (green-red), and *b*∗ (yellow-blue) were recorded based on the CIELAB system (directional annular 45° illumination, D65 daylight mode).

#### 2.3.6. Scanning Electron Microscopy

Scanning electron microscopy was conducted for flour and biscuit samples. For microstructure evaluation, samples, after first being cut into specific dimensions (20 × 20 mm), were defatted with hexane at a ratio of 1 : 5. The samples were mounted on cylindrical samplers using conductive adhesive and coated with gold using a vacuum pump. The microstructure structure of the flour and biscuits (surface and vertical cross-section) was examined with a scanning electron microscope (JEOL, Model JSM-5310, JEOL Ltd., Japan), using an accelerating potential of 15 kV.

#### 2.3.7. Sensory Evaluation

The sensory evaluation was conducted using a 10-member trained panel consisting of students and staff, both males and females, age 22–45 years old, from the Department of Food Science and Technology of the University of West Attica in Athens, Greece, as previously described by Lazou et al. [28, 30]. Panelists, after they had been trained in the specific vocabulary and methodology of sensory analysis according to ISO 13299 : 2016 [31], participated in the descriptive analysis of the biscuits. The samples were presented in three-digit-coded identical white dishes at room temperature in a sensory evaluation lab equipped with separate partitioned booths, ensuring a controlled climate and normal lighting. Sessions were of 19 45 minutes, in each of which four samples were presented and, in a manner, permitted three replicates for each sample. Between samples, water was used by panelists to clean their palates. The five main categories of descriptive terms were as follows: appearance, odor, flavor, taste, and texture. A 9-point scale ranging from 1 = lower intensity/imperceptible characteristic, 5 = quite perceptible characteristic, and 9 = higher intensity/extremely perceptible/very intense was used.  Table 2 sums up the sensory attributes, definitions, and anchors that have been used in this evaluation [32].

The overall sensory acceptability was performed as previously described by Lazou et al. [30], using 50 untrained panelists, both men and women, consisting mainly of graduate students and staff members of the Department of Food Science and Technology of the University of West Attica in Athens, Greece, and according to the experimental design, in a manner permitting the evaluation of three replicates. A nine-point hedonic scale ranging from 1 (dislike extremely) to 9 (like extremely) was used for the evaluation of biscuits [28, 30].

### 2.4. Experimental Design

This was a 2 (chickpea or lentil flour) × 2 (sweetener content) × 3 (legume/wheat flour ratio) full factorial experimental design with two replications. The independent variables of the experiment were the type of legume flour (chickpea, lentil), the oligofructose content (0, 50, and 100%), and the proportion of legume flour substitution (10, 15, and 30%). This resulted in 12 independent samples. Both the level and the sample coding are summarized in Table 1(b).

### 2.5. Fitting and Statistical Analysis

The results obtained in the present study were subjected to a one-way analysis of variance (ANOVA) with Duncan's hoc test in order to reveal the effect of sugar and legume flour substitution. Furthermore, principal component analysis was implemented to investigate correlations between instrumental and sensory characteristics and find possible groups of biscuits in terms of quality characteristics. For all analyses, the statistical program Statistica 7 was used to evaluate the statistical significance of the data.

## 3. Results and Discussion

The physicochemical properties of the innovative legume-based biscuits with alternative sweeteners are shown in Table 3. The moisture content of various biscuit formulations ranged between 2.11 ± 0.07% (w.b.) (BC31) and 6.10 ± 0.08% (w.b.) (BC32). The addition of oligofructose in whole-meal dicoccum wheat biscuit formulations caused an increase in moisture content. However, the oligofructose level did not lead to significant moisture content differences due to its humectant properties and its higher water retention capacity [33]. Chickpea and lentil flour additions affected moisture content in a complex way, causing fluctuating moisture content values depending on the levels of oligofructose and legume flour (Table 3). It could be said that the water activity values of the final biscuit samples generally followed the moisture content, ranging between 0.247 ± 0.00 (BC31) and 0.498 ± 0.01 (B13) (Table 3). The water activity of chickpea- and lentil-containing biscuits was within the range reported in the past [13, 34–36] and was suitable to ensure long shelf life and textural attributes like crispness.

**Figure 2:**
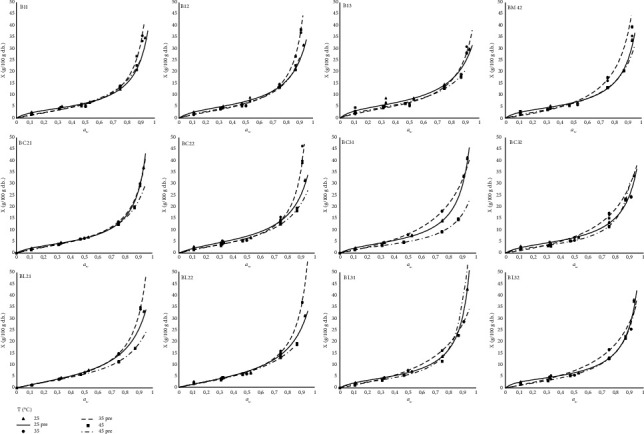
Sorption isotherms of innovative legume-based biscuits with alternative sweeteners.

The sorption isotherms of the innovative legume-based biscuits with alternative sweeteners were described by the GAB model. The results of parameter estimation and *R*^2^ are summarized in Table 4, while in Figure 2, the experimental vs. predicted values of isotherms are presented. It can be seen that all biscuits, prepared using different formulations, exhibited the characteristic S-shaped isotherms typical for cereal-based products. The values of the predicted monolayer moisture content *X*_m_ ranged from 3.039 to 9.095% d.b. among different samples and temperatures. Guiné et al. [35] reported values of 4.95 and 19.81% d.b. at 25°C and 5.29 and 19.60% d.b. at 40°C for Maria biscuits from different brands showing higher values for some commercial bands, being concurrently quite different from the values determined for control samples in the present study, while Hao et al. [37] reported values of 4.428%, 4.393%, and 4.210% for biscuits prepared using different levels of corn oil. Panjagari et al. [38] using the GAB model reported *X*_m_ values ranging from 2.66% at 37°C to 1.29% at 45°C for biscuits rich in beta-glucan, showing the effect of increased fiber presence. The increase in temperature resulted in a decrease in the equilibrium moisture content of the samples. This might be explained by how the excitation state and mobility of water molecules are affected by temperature; when temperature rises, the attractive forces weaken as a result of an increase in the distance between the adsorbed and the sorption site, which results in a reduction in moisture content. The addition of legume flour resulted in increased values of *X*_m_ in the biscuits compared with the control. The same effect was exhibited with the addition of oligofructose to the formulations. These variations can be explained by the changes in the preferred sites of absorption and, consequently, the water binding capacity due to the increase in protein and fiber levels in the formulation. The values of the *k* parameters for the biscuits are very close to one (Table 4). This trend indicates that the water molecules beyond the monolayer are not structured into a multilayer but have the same characteristics as the molecules in the bulk liquid [29, 39]. GAB model parameter *k* close to one has also been reported by Palou et al. [40] for different cookies, Hao et al. [37] for fermented biscuits, and Panjagari et al. [38] for beta-glucan-rich composite flour biscuits. The enthalpic parameter values of the GAB model, *C*, exhibited lower values for lentil flour-containing formulations. Further, it should be noted that biscuit formulations contained legume flour, which increased the protein and fiber content of biscuits along with oligofructose. This probably led to an increase in the complexity of the system. For example, supplementary foods containing cereals and legumes showed a steep rise above 64% RH [41]. Moreover, Moreno-Vilet et al. [42] found that the hygroscopicity of inulin is high at moisture retention up to 35% (wet basis) (*a*_w_ 0.5-0.9), while at high *a*_w_ (0.9), it retains little water; this might depend on the inulin state and drying method as well. Schaller-Povolny et al. [43] reported that the sorption isotherm behavior of the inulin depends on molecular weight distribution, with low-molecular-weight inulins absorbing less water than those of higher molecular weight, which offer easier access to binding sites. This caused legume- and oligofructose-containing biscuit isotherms to be close in the temperature range studied and to show a crossing over or intersection at *a*_w_ higher than 0.5-0.7 (Figure 2).

The structural and textural properties of biscuits prepared using substitutions for chickpea and lentil flour, as well as oligofructose, are given in Table 5. As can be seen, the spread ratio of biscuits differed significantly depending on legume type, legume, and oligofructose substitution among different formulations. The spread ratio mean values of lentil-containing biscuits were the highest, while the lowest was for the mixture-containing product. The increase in legume flour proportion led to a significant increase in spread ratio, while the addition of oligofructose had the opposite effect. Generally, oligofructose addition results in an increase in biscuit spread ratio due to its higher solubility, as it shows increased affinity for water compared to sucrose [33]. However, the addition of oligofructose, though leading to an increase in biscuits made with whole-meal dicoccum wheat flour, led to a reduction in the spread ratio, probably due to the simultaneous presence of oligofructose and legume flour. Such an effect has been observed by Hajas et al. [24] in wheat-lentil-wheat protein cookies. It should be noted that oligofructose progressively dissolves during baking, resulting in an increase in spread ratio and acting as a fat enhancer [44, 45]. Substitution of whole-meal dicoccum wheat flour with 15% chickpea and 15% lentil flour along with 50% oligofructose (BM42) had no significant effect on the spread ratio compared to biscuits without any substitution. Furthermore, it should be noted that chickpea proteins have good functional qualities (solubility, water and oil absorption capacity, emulsifying, foaming, and gelling properties), which are strongly dependent on the protein profile [46]. The partial substitution of whole-meal dicoccum wheat flour with chickpea flour increases protein content while also altering the rheological and functional aspects of biscuits. Such an addition can also lead to a reduction in the glycemic index. Similarly, the addition of lentil flour in biscuit formulations can affect the spread ratio. The protein-rich and fiber-packed nature of lentil flour can contribute to spread ratio changes, either increasing or decreasing depending on the presence of oligofructose. Lentil flour also has good functional properties that can affect or influence the properties of biscuits through various mechanisms. It should be noted that amino acid composition and sequence, net charge, and hydrophobicity determine the functionality of lentil flour proteins, which is also affected by starch functionality depending on the amylose and amylopectin ratios as well as their physical structure [47, 48]. It is derived that the right balance during biscuit formulation could lead to biscuits with a desirable spread and appealing texture, thus contributing to better sensory acceptability.

The analysis of variance showed that all the independent variables affected the apparent density of the biscuits. Due to the composition of chickpea and lentil flours, which affect the matrix of each product, the apparent density ranged from 344 ± 16 kg/m^3^ (BC32) to 515 ± 42 kg/m^3^ (BL21) (Table 5), describing the matrix compactness of the products. Generally, the addition of legume flour resulted in an increase in the apparent density mean value of most chickpea or lentil flour at the 10% incorporation level, while the sugar substitution had no significant. Contributors to the variations in apparent density might also be the moisture content and particle size of legume flour [49, 50]. The true density of the biscuits exhibited significant differences only among plain whole-meal dicoccum wheat samples and chickpea, lentil, and chickpea-lentil substitutions, while the level of substitution within the same legume group had not exerted any significant effect (Table 5). It should be noted that chickpea and lentil flours are relatively coarse. Their incorporation into the biscuits might also partially explain the differences in their behavior due to the change in water absorption capacity [51], which can affect porosity through the creation of an open structure in biscuits, leading to a high number of pores. Porosity values for all biscuits are shown in Table 5. As can be seen, the addition of oligofructose significantly reduced the porosity of the control samples. Further, the addition of both chickpea and lentil flours also led to a reduction in porosity compared to B11 biscuits. Generally, the porosity values of biscuits varied between 0.647 ± 0.01 (BL21) and 0.76 ± 0.01 (BC32). It seems that the addition of chickpea and lentil flours, along with oligofructose, causes significant porosity changes in a complex way. This could be attributed to the reduction of gluten proteins, the presence of legume proteins, increased fiber, and increased starch gelatinization during baking. The swelling of cells and starch granules can lead to porosity reduction [52]. Further, the increased aeration generally leads to higher porosity values in the baked biscuits. Also, increased fat levels also help in increased air incorporation during creaming, resulting in biscuits with increased porosity [53, 54]. The addition of oligofructose, which can act as a fat enhancer, might have a similar effect.

Table 5 also depicts the instrumental hardness (breaking strength) of the innovative biscuits. The oligofructose substitution for sugar in control biscuits caused a significant increase in *F*_max_, while the addition level did not result in significant differences. In the past, an opposite effect has been reported [33]. This increase could be attributed to the fat-enhancing properties of oligofructose, which could lead to an increase in biscuit hardness [25, 55]. The addition of lentil flour caused a significant increase in biscuits' *F*_max_, with the highest value observed for the samples containing 15 and 30% legume flour, independently of the sugar substitution. Legume flour addition impacts the texture of biscuits by influencing parameters such as hardness, crumb structure, and chewiness. Both flours with fibrous natures can result in a denser and more compact crumb structure, leading to increased hardness. Legume-based biscuits often exhibit increased hardness due to lower gluten formation. Legumes contain proteins that can interact with wheat proteins in the biscuit dough. The proteins in legumes, such as globulins and albumins, can contribute to the formation of a protein network [11, 18, 20, 24, 46, 56]. This network affects the dough's viscoelastic properties, leading to changes in the microstructure of the baked biscuits. It can influence factors like crumb texture, moisture retention, and overall product quality. The increased fiber incorporation through chickpea and lentil flour addition could also account for modified dough behavior and biscuit properties, while fiber type also contributes to the extent of these effects [44]. It should be noted that careful formulation and the use of appropriate processing techniques, such as adjusting moisture content and leavening agents, are also crucial for achieving desired textural characteristics.

The biscuits' color is a crucial parameter influencing their acceptability. The comparison of color properties among different biscuit formulations is shown in Table 3. All the independent variables caused significant differences in color among the biscuit formulations. The *L*∗ parameter values decreased with the oligofructose addition, while the *a*∗ and *b*∗ parameters increased, indicating the level of browning and confirming that both red coloring and yellow coloring are dominating over the green due to whole-meal wheat fiber [4, 57]. Also, this might be attributed to the elevated baking temperature, the type of oligofructose used, and its ability to participate in browning reactions [58]. The addition of legume flour decreased the lightness of the products and increased the *a*∗ and *b*∗ parameters. Chickpea addition increases protein content, which increases the reaction between reducing sugars and amino acids, leading to an increase in redness (*a*∗) [59]. An analogous behavior has been reported by Koukoumaki et al. [18] for crackers prepared by replacing wheat flour with chickpea flour. Lentil-containing biscuits show physical attributes depending on a combination of interactive factors, among which are the high phenolic content, melanoidins formed through browning reactions, the high protein and fiber content, and the reducing sugars, sugar, and oligofructose presence [11, 24, 48, 60]. The total color difference (Δ*E*) for all biscuit formulations was significantly affected by independent variables in a pattern that followed the *L*∗ value changes (Table 3). It should be noted that Δ*E* generally represents the darkness or lightness variation in biscuits. The lowest value for Δ*E* was observed in biscuits containing 10% lentil flour (BL21) and the highest in samples containing 15% chickpea, 15% lentil, and 50% oligofructose (BM42) (Table 3).

**Figure 3:**
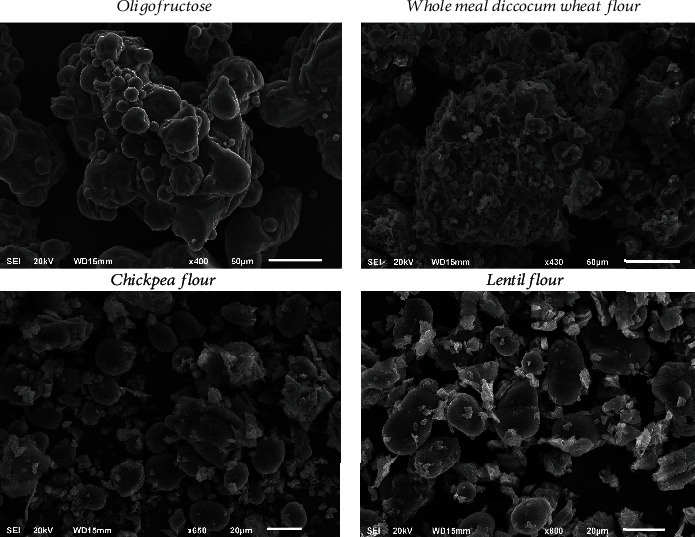
SEM images of raw material (oligofructose, whole-meal dicoccum wheat flour, chickpea flour, and lentil flour).

The incorporation of legume flours in biscuit formulations affects the microstructure and subsequent quality attributes. The size, form, and concentration of legume particles along with whole-meal dicoccum wheat flour influence the distribution pattern within the biscuit. These particles can impact the overall particle distribution and create heterogeneity within the biscuit matrix, affecting the visual appearance of the microstructure. In Figure 3, SEM images of biscuit formulation raw materials (whole-meal dicoccum wheat flour, chickpea flour, lentil flour, and oligofructose) are shown. A SEM image of oligofructose revealed that it was agglomerated, showing a spherical morphology. It should be pointed out that inulin morphology depends on water activity, and in medium *a*_w_, it is randomly arranged, while in high *a*_w_, it has a needle-like morphology [61]. The SEM image of the whole-meal dicoccum wheat flour showed two types of starch granules, which appear to have oval shapes with some irregularities, probably due to their spatial disposition and their compacted arrangement within the grain, protein bodies, and fiber particles as well. This confirms findings reported in the past [62–64]. The chickpea flour SEM image depicts the coarse character of this flour, along with a higher number of protein bodies. This was expected, as chickpeas exhibit a higher protein content. Further, starch granules appeared to be smaller and oval, along with fiber fragments. Similar findings have also been reported by Almusallam et al. [63] and Zafar et al. [64]. Lentil flour was also coarse in nature, containing more protein and fiber as well. As can be seen from Figure 3, starch granules were agglomerated and/or linked to fiber and other constituents, while their shape appeared to be round, oval, or irregular. This might be attributed to the grinding process in the stone mill. Similar findings have been reported by Ahmed and Nahar [65] and Benmeziane-Derradji et al. [62].

**Figure 4:**
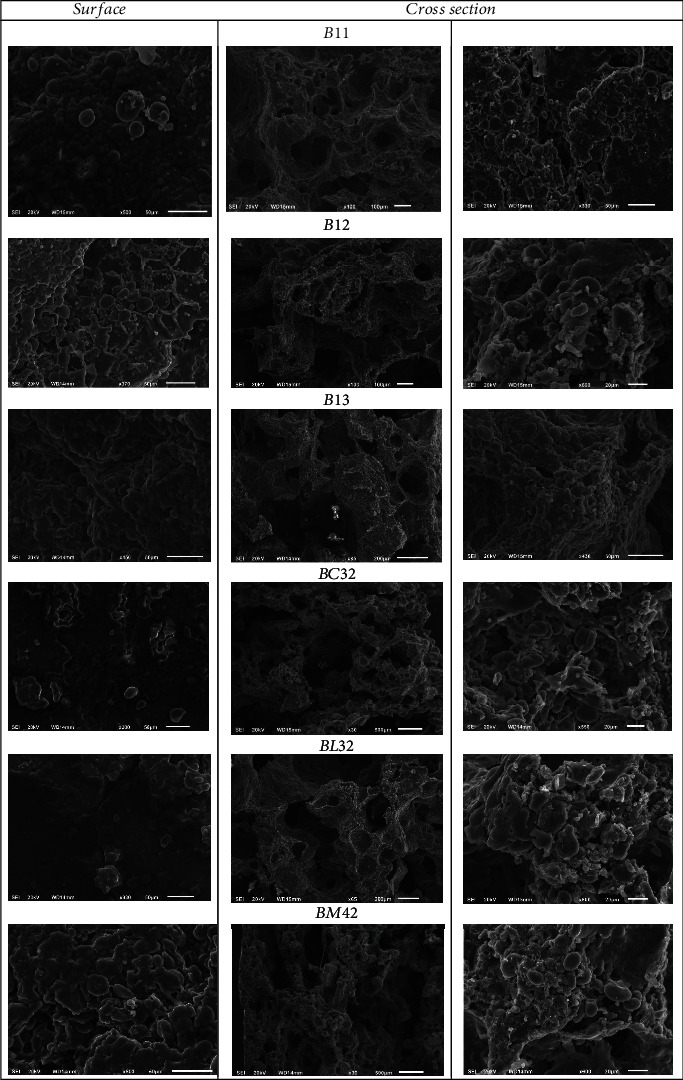
SEM images of surface and cross-section of innovative legume-based biscuits with alternative sweeteners.

In Figure 4, the surface microstructures and the internal (cross-section) microstructure of all biscuit's formulations are shown. For the control sample (B11), the surface shows very small pores along with some big pores and crevices. Its cross-section SEM images show that starch granules are embedded within the protein matrix. Also, it appeared that starch granules largely kept their form (especially the small granules); this could be attributed to hampered gelatinization due to restricted water availability and high sugar and fat content [66]. It should be noted that the incomplete starch gelatinization leads to decreases in water absorption, causing the biscuits to show a harder texture [67]. Further, air cavities were present, due mainly to water vapor formation during baking, which also led to biscuit expansion. The microstructure of the surface and cross-section of the chickpea flour and oligofructose-containing biscuits BC32 are shown in Figure 4. The biscuit surface appeared to host a rather high number of holes and crevices, along with fiber fragments, while the starch granules were embedded in the protein matrix, as the chickpea addition resulted in a protein increase. Cross-section SEM images better show the porous structure of the biscuit, as well as the fact that starch granules were embedded within the protein matrix, having undergone restricted gelatinization, keeping to a great degree their form existing in different sizes, probably due to their different origin, along with oligofructose granules. As chickpea addition leads to increased protein and the presence of high fat in the biscuits produced, an enhanced entrapment of starch was observed. A similar effect has been reported by Lu et al. [68], Saeed et al. [69], and Singh Sibian and Singh Riar [70], while Almusallam et al. [63] reported that chickpea flour addition leads to the development of a sheet-like structure, which entraps the majority of starch granules, while some protein bodies seem to be intact. The biscuit structure changes due to the chickpea flour addition have a significant influence on its properties, as has already been mentioned and previously reported by AL-Ansi et al. [71]. SEM images of biscuits containing 30% lentil flour along with 50% oligofructose (BC32) revealed more and larger irregular holes and crevices on the surface as well as in cross-section. Starch and oligofructose granules were entrapped mainly in the protein matrix, including fiber stripes, as lentil flour contains more protein and fiber. Starch granules appeared to largely keep their shape intact, also due to the hampering of gelatinization due to the presence of less water. The surface and cross-section of biscuits containing 15% chickpea and 15% lentil, along with 50% oligofructose (BM42), exhibited a similar microstructure with relatively large and numerous pores, showing high porosity values as well.

**Figure 5:**
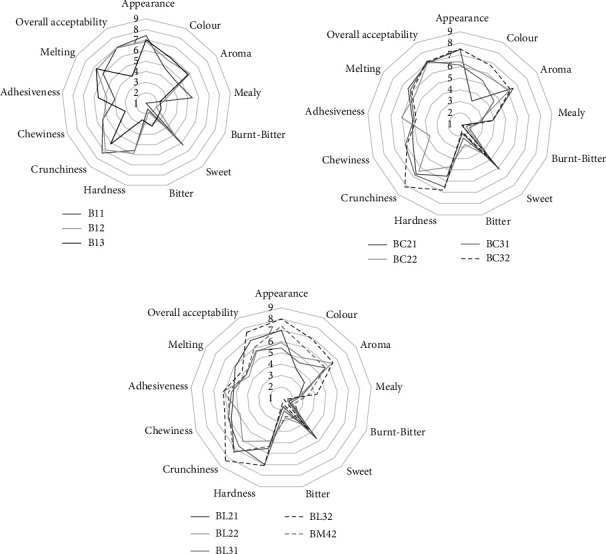
Sensory characteristic variations of innovative legume-based biscuits with alternative sweeteners.

Figure 5 shows the descriptive sensory attributes scores for all biscuit formulations along with their overall acceptability. The analysis of variance revealed that the independent variables did not have a significant effect on the mean value of appearance of all biscuits, indicating that all the formulations had an appealing appearance without any defects. The products' color and aroma were affected mainly by oligofructose addition, which led to the formation of darker products but did not affect biscuit appearance while significantly affecting color and aroma scores. The aroma of biscuits with oligofructose was more intense. The addition of legume flour to the formulations led to an increase in mealy flavor. Oligofructose at 50 and 100% substitution gave less mealy biscuits. Generally, burnt-bitter scores were low, though oligofructose caused a significant increase in their mean values (B12, B13). As expected, sugar substitution at 100% with oligofructose resulted in biscuits with reduced sweetness. Organoleptic hardness was significantly reduced by the 100% sweetener substitution, while no differences were detected at the 50% substitution with the control sample. A significant effect also occurred with the legume flour addition to the formulation. The harder samples were observed at addition levels of 30%, with the lentil-containing biscuits having higher mean values. Crunchiness was also affected by oligofructose addition at a level of 100%, while at 50%, no significant differences were observed with the control formulation. Moreover, crunchiness values increased with the addition of legume flour. A significant decrease in the chewiness of biscuits was recorded by total sugar substitution with oligofructose (B13), while the legume flour addition had the opposite effect. Adhesiveness was significantly increased in biscuit formulations containing 100% oligofructose. The melting of the biscuits was significantly affected by the legume flour type and its addition ratio. The formulations with lentil flour addition exhibited lower melting values, as well as those with legume addition higher than 10%.

As described by untrained panelists, oligofructose gave biscuits with lower overall acceptability, receiving a very low score of 3.9 ± 0.8 for the B13 formulation. From the independent parameters, the legume type and the sweetener substitution significantly affected the acceptability of the biscuits. The mean values of acceptability of chickpea-containing formulations were higher, while products containing 50% oligofructose were classified in the same group as the control sample, indicating that they had an equally high acceptance score. The addition of legume flour at a level of 10 and 30% resulted in higher mean scores of overall acceptance of these biscuits. Chickpeas containing biscuits both with sugar and/or oligofructose (BC21, BC22, BC31, and BC32) were equally acceptable to the control formulation. Also, biscuits containing 30% lentil and oligofructose at a level of 50% (BL32) showed no difference from the control. Additionally, the formulations that exhibited the highest score of overall acceptability and were similar to the control samples (≥7) were all the chickpea flour-containing formulations, as well as the 30% lentil with oligofructose. The characteristics of these biscuits are that they had good appearance, color, aroma, and flavor; their sweetness had a score higher than 5; they were crunchy; they were moderated in hardness and chewiness; and they had desired hydration behavior during the mastication process (melting). Similarly, high overall acceptability of biscuits produced using wheat and lentil at a 93 : 07 ratio has been reported by Saleem et al. [23]. AwadElkareem and Shammari [72] also reported good acceptability for biscuits prepared with 5 and 10% lentil, with samples at the 5% level receiving the highest scores. Hajas et al. [24] found that the overall liking of cookies prepared using red and brown lentils was higher compared to black lentil cookies, while no differences were detected between green, red, and yellow lentil cookies. Good overall acceptability has also been reported for biscuits containing 10% and 20% chickpea [73]. Rababah et al. [59] reported no significant differences between biscuits containing 3% chickpea as well as 3% soy protein isolate or 12% broad bean. Recently, Talens et al. [74] reported that biscuits prepared using 50 : 50 rice and chickpea were awarded scores ≥ 6 (out of 9) by 52% of adolescents for their overall quality, with 24% of them describing the product as having a “biscuit” and 12% as having a “nutty” flavor, while 55% of the participants could not be able to notice any dominant flavor.

**Figure 6:**
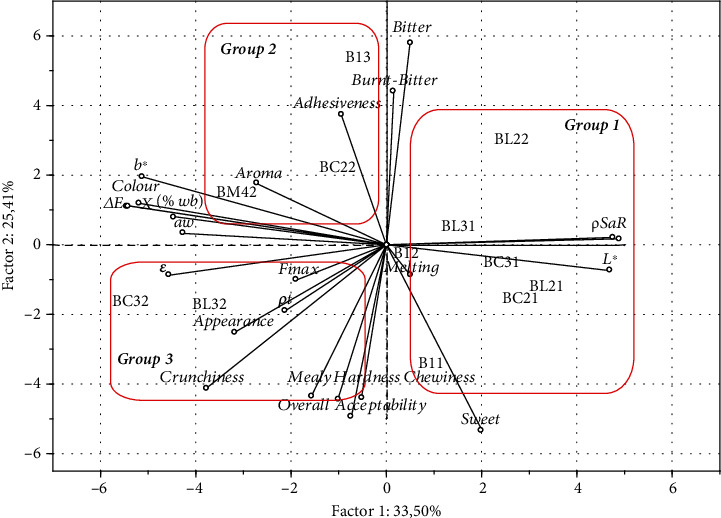
Principal component analysis results.

In order to more closely reveal the effect of sweetener and legume flour additions on the quality characteristics of the biscuits, the physicochemical, structural, and textural characteristics, as well as sensory attributes, were subjected to principal component analysis. Analysis revealed that the first 3 principal components accounted for 72.93% of the total variability, with 58.91% explained by the first 2.  Figure 6 shows the biplot of the first 2 PCs, factor 1 and factor 2, each of which accounted for 33.50% and 25.41%, respectively, of the total variability. The results indicated that the spread ratio, apparent density, and *L*∗ were positively correlated, while these properties were negatively correlated with moisture content, water activity, aroma, and color difference, both instrumental and sensory. The latter properties were highly correlated, indicating that the increase in biscuit moisture content leads to an increase in water activity and the sensory color of them. Moreover, the porosity of the biscuits was negatively correlated with the apparent density and spread ratio. This fact explains and supports the phenomenon that the increased spread of biscuits corresponds to increased apparent density and lightness and decreased porosity. The pore formation happens inside the dough during the baking process, leading to the spread of the biscuits. There was a positive correlation between textural characteristics (both instrumental and sensory) and appearance, and they also positively correlated with overall acceptability. The crunchier, harder, and chewier the biscuits, the better their appearance and acceptability become. The texture, as was expected, is the key quality characteristic for the acceptance of legume-based biscuits prepared using alternative sweeteners. On the other hand, there was a negative correlation between acceptability, textural properties, and bitter taste and flavor, indicating that acceptable samples exhibit good chewing characteristics and are not bitter. In contrast, they were relatively sweet, as sweet taste correlated negatively with bitter flavor. In Figure 6, 3 main groups of biscuits are indicated. Group 1 is characterized by a high spread ratio, apparent density, lightness, and low moisture content and aroma. These samples contain chickpea and lentil flour without oligofructose substitution. Group 2 is characterized by a high bitter taste and flavor, low textural attributes, and, consequently, low acceptability. These are the samples containing 10% chickpea flour and 50% oligofructose and the sample with wheat flour and 100% oligofructose. Group 3 samples are characterized by their good textural characteristics, good appearance, and high acceptability. These samples contain chickpea and lentil flour at a 30% ratio, and the sugar has been substituted at a 50% level with oligofructose. It has to be mentioned that the sample containing a mixture of legume flour and oligofructose exhibited high aroma values and moderate textural attributes. From the preceding analysis, it can be revealed that the most acceptable biscuits were those that contained oligofructose, lentil, and chickpea flour up to a 30% ratio.

## 4. Conclusions

The findings have demonstrated that by substituting Triticum dicoccum whole-meal flour with chickpea or lentil flours and oligofructose, it is feasible to produce highly acceptable, innovative legume-based high-protein biscuits containing 50% oligofructose and lentil or chickpea flour up to a 30% ratio. The property correlation, both physical and sensory, revealed that the most important quality characteristic for the acceptance of legume-based biscuits prepared using alternative sweeteners was texture and sweet taste. The use of dicoccum wheat flour and its enrichment with legumes and alternative sweeteners would further ensure increased utilization of the legumes in products widely consumed by consumers. The results on the properties received could be the basis for further study and help in developing commercial formulations with multiple substitutions.

## Figures and Tables

**(a):** 

*Oligofructose substitution*				
Ingredient	0% (control)		50%	100%
Flour, dicoccum	100		100	100
Sugar	26.2		13.1	0
Shortening	21.4		21.4	21.4
Baking powder	1.3		1.3	1.3
Baking soda	1.3		1.3	1.3
Water	20		20	20
Oligofructose	0		13.1	26.2

*Legume substitution*				
Ingredient	10%		30%	
Flour, dicoccum	90		70	
Flour, legume (lentil or chickpea)	10		30	
Sugar	13.1		0	
Shortening	21.4		21.4	
Baking powder	1.3		1.3	
Baking soda	1.3		1.3	
Water	20		20	
Oligofructose	0		0	

*Legume and oligofructose substitution*				
Ingredient	10%	30%	15-15%	
Flour, dicoccum	90	70	70	
Flour, legume (lentil or chickpea)	10	30	15	
Sugar	13.1	13.1	13.1	
Shortening	21.4	21.4	21.4	
Baking powder	1.3	1.3	1.3	
Baking soda	1.3	1.3	1.3	
Water	20	20	20	
Oligofructose	13.1	13.1	13.1	

**(b):** 

	Legume flour type			*C*_F_, flour substitution (legume/wheat %)				*C*_S_, sweetener content (oligofructose/sugar %)		
Values	Chickpea	Lentil	Chickpea/lentil	0	10	15	30	0	50	100
Coding	BC	BL	BM	1	2	4	3	1	2	3

Coding example: 1^st^ letters: type of legume flour. BC12: biscuit with 0% legume/wheat ratio, with 50% oligofructose; BL32: biscuit with 30% lentil/wheat ratio, with 50% oligofructose.

**Table 2:** Terms, attribute definitions, and anchors used in the descriptive analysis of biscuits.

Attribute	Description	Intensity
*Appearance*	Uniformity of appearance (shape, presence of cracks or holes)	Low-high
*Color*	Basic coloring	Light-dark
*Smell*	Overall aroma intensity	Low-high
Aroma		
*Flavor*		
Mealy	Raw flour	Low-high
Burnt-bitter	Burnt sample	Low-high
*Taste*	Fundamental taste sensation of which sucrose is typical	Low-high
Sweet		
Bitter	Residual taste sensation of which caffeine in water is typical	Low-high
*Texture*		
Hardness	Force exerted during the first bite of the sample	Low-high
Crunchiness	Degree of noise released during chewing with the molar tooth	Low-high
Chewiness	Difficulty during chewing	Low-high
Adhesiveness	Sample adhesion during chewing or to other surfaces and creating a sticky mass/degree to which particles of a sample stick together	Low-high
Melting (hydration and dissolution)	Quantity dissolved	Low-high

**Table 3:** Physicochemical properties of innovative biscuits as affected by legume addition and sugar substitution.

Coding	Material	*C*_F_ (legume/wheat %)	*C*_S_ % (oligofructose/sugar %)	*a*_w_	*X* (% w.b.)	*L*∗	*a*∗	*b*∗	Δ*E*
B11	Dicoccum flour	0	0	0.409 ± 0.03^c^	3.696 ± 0.10^b^	64.82 ± 0.99^d^	6.00 ± 0.54^a^	26.56 ± 0.31^a^	—
B12	Dicoccum flour	0	50	0.488 ± 0.02^d^	4.424 ± 0.16^c^	64.86 ± 0.90^d^	6.99 ± 0.09^a^	30.61 ± 0.29^b^	4.20 ± 0.30^b^
B13	Dicoccum flour	0	100	0.498 ± 0.01^d^	4.784 ± 0.08^c^	62.55 ± 0.48^c^	8.98 ± 0.54^c^	31.98 ± 1.03^c^	7.01 ± 1.01^c^
BC21	Chickpea	10	0	0.341 ± 0.01^b^	3.812 ± 0.11^b^	64.78 ± 3.16^d^	6.76 ± 1.10^a^	27.63 ± 3.78^a^	4.24 ± 1.96^b^
BC22	Chickpea	10	50	0.338 ± 0.00^b^	4.329 ± 0.10^c^	61.36 ± 1.12^b^	10.15 ± 1.00^d^	35.73 ± 1.97^e^	8.98 ± 1.50^d^
BC31	Chickpea	30	0	0.247 ± 0.00^a^	2.110 ± 0.07^a^	65.90 ± 1.79^d^	7.20 ± 0.59^b^	30.48 ± 1.57^b^	4.35 ± 1.93^b^
BC32	Chickpea	30	50	0.536 ± 0.00^d^	6.102 ± 0.08^d^	57.88 ± 0.49^a^	13.24 ± 0.44^e^	36.38 ± 0.36^f^	14.04 ± 0.58^e^
BL21	Lentil	10	0	0.290 ± 0.01^a^	2.962 ± 0.09^a^	63.16 ± 0.56^d^	6.10 ± 0.30^a^	25.78 ± 0.24^a^	1.77 ± 0.33^a^
BL22	Lentil	10	50	0.300 ± 0.01^b^	3.732 ± 0.10^b^	62.16 ± 0.44^c^	8.47 ± 0.48^c^	29.21 ± 0.16^b^	4.65 ± 0.49^b^
BL31	Lentil	30	0	0.272 ± 0.03^a^	2.372 ± 0.06^a^	60.08 ± 0.66^b^	8.12 ± 0.31^c^	29.47 ± 0.20^b^	5.97 ± 0.48^c^
BL32	Lentil	30	50	0.398 ± 0.01^c^	4.065 ± 0.12^b^	57.66 ± 0.87^a^	12.27 ± 0.64^e^	33.21 ± 0.19^d^	11.83 ± 0.44^d^
BM42	Lentil-Chickpea	15	50	0.414 ± 0.01^c^	4.233 ± 0.09^c^	57.73 ± 1.31^a^	12.71 ± 0.87^e^	35.19 ± 0.21^e^	13.07 ± 1.26^e^

Samples with different letters in the same column differ significantly (*p* <0.05).

**Table 4:** GAB parameters estimation of innovative legume-based biscuits with alternative sweeteners.

Coding	Material	*C*_F_ (legume/wheat %)	*C*_S_ % (oligofructose/sugar %)	*T* (°C)	*X*_m_	*K*	*C*	*R* ^2^
B11	Dicoccum flour	0	0	25	3.757 ± 0.12	0.949 ± 0.00	10.751 ± 2.74	0.999
B11	Dicoccum flour	0	0	35	3.855 ± 0.29	0.983 ± 0.01	4.161 ± 1.65	0.999
B11	Dicoccum flour	0	0	45	5.352 ± 0.77	0.898 ± 0.03	2.395 ± 0.74	0.997
B12	Dicoccum flour	0	50	25	4.421 ± 0.12	0.917 ± 0.00	9.851 ± 1.74	0.999
B12	Dicoccum flour	0	50	35	3.991 ± 0.25	0.986 ± 0.01	4.189 ± 1.32	0.999
B12	Dicoccum flour	0	50	45	4.177 ± 0.31	0.939 ± 0.01	5.327 ± 1.50	0.998
B13	Dicoccum flour	0	100	25	4.960 ± 0.15	0.889 ± 0.01	12.214 ± 2.53	0.999
B13	Dicoccum flour	0	100	35	4.342 ± 0.33	0.935 ± 0.01	5.506 ± 1.93	0.997
B13	Dicoccum flour	0	100	45	4.860 ± 0.44	0.859 ± 0.02	6.836 ± 2.22	0.997
BC21	Chickpea	10	0	25	3.688 ± 0.05	0.963 ± 0.00	8.939 ± 0.95	0.999
BC21	Chickpea	10	0	35	4.270 ± 0.13	0.946 ± 0.00	4.313 ± 0.54	0.999
BC21	Chickpea	10	0	45	4.797 ± 0.22	0.893 ± 0.01	3.331 ± 0.38	0.999
BC22	Chickpea	10	50	25	4.827 ± 0.35	0.906 ± 0.01	5.704 ± 1.93	0.996
BC22	Chickpea	10	50	35	3.815 ± 0.23	0.995 ± 0.01	4.933 ± 1.75	0.998
BC22	Chickpea	10	50	45	5.066 ± 0.61	0.868 ± 0.03	3.143 ± 0.88	0.996
BC31	Chickpea	30	0	25	4.060 ± 0.07	0.965 ± 0.00	8.028 ± 0.92	0.999
BC31	Chickpea	30	0	35	9.095 ± 0.56	0.848 ± 0.01	1.476 ± 0.15	0.999
BC31	Chickpea	30	0	45	3.140 ± 0.11	0.910 ± 0.01	7.157 ± 0.99	0.999
BC32	Chickpea	30	50	25	3.979 ± 0.18	0.939 ± 0.01	9.621 ± 3.09	0.998
BC32	Chickpea	30	50	35	8.000 ± 2.09	0.862 ± 0.04	1.358 ± 0.59	0.994
BC32	Chickpea	30	50	45	3.039 ± 0.11	1.001 ± 0.01	7.751 ± 1.66	0.999
BL21	Lentil	10	0	25	5.571 ± 0.11	0.900 ± 0.00	2.764 ± 0.17	0.999
BL21	Lentil	10	0	35	4.838 ± 0.14	0.952 ± 0.00	2.874 ± 0.28	0.999
BL21	Lentil	10	0	45	4.656 ± 0.27	0.862 ± 0.01	3.657 ± 0.51	0.999
BL22	Lentil	10	50	25	5.648 ± 0.70	0.884 ± 0.02	2.557 ± 0.88	0.996
BL22	Lentil	10	50	35	4.615 ± 0.34	0.968 ± 0.01	3.133 ± 0.84	0.998
BL22	Lentil	10	50	45	6.387 ± 1.12	0.822 ± 0.04	2.070 ± 0.57	0.996
BL31	Lentil	30	0	25	3.773 ± 0.02	0.975 ± 0.00	3.657 ± 0.51	0.999
BL31	Lentil	30	0	35	8.700 ± 0.30	0.826 ± 0.01	1.473 ± 0.08	0.999
BL31	Lentil	30	0	45	3.209 ± 0.13	0.991 ± 0.01	5.433 ± 1.06	0.999
BL32	Lentil	30	50	25	3.666 ± 0.11	0.962 ± 0.00	14.459 ± 4.25	0.999
BL32	Lentil	30	50	35	7.767 ± 1.26	0.855 ± 0.03	1.654 ± 0.48	0.997
BL32	Lentil	30	50	45	4.355 ± 0.29	0.938 ± 0.01	2.701 ± 0.50	0.998
BM42	Lentil-chickpea	15	50	25	3.876 ± 0.10	0.943 ± 0.00	11.820 ± 2.39	0.999
BM42	Lentil-chickpea	15	50	35	6.274 ± 1.05	0.942 ± 0.02	1.495 ± 0.60	0.996
BM42	Lentil-chickpea	15	50	45	4.877 ± 0.41	0.898 ± 0.02	3.235 ± 0.70	0.998

**Table 5:** Structural and textural properties of innovative biscuits as affected by legume addition and sugar substitution.

Coding	Material	*C*_F_ (legume/wheat %)	*C*_S_ % (oligofructose/sugar %)	SR	*ρ*_a_ (kg/m^3^)	*ρ*_t_ (kg/m^3^)	*ε*	*F*_max_ (N)
B11	Dicoccum flour	0	0	7.92 ± 0.23^b^	355 ± 15^a^	1401 ± 10^a^	0.749 ± 0.01^c^	6.97 ± 0.91^a^
B12	Dicoccum flour	0	50	8.34 ± 0.19^c^	379 ± 15^a^	1401 ± 10^a^	0.734 ± 0.01^b^	8.20 ± 0.45^b^
B13	Dicoccum flour	0	100	7.07 ± 0.26^b^	393 ± 23^a^	1401 ± 10^a^	0.713 ± 0.01^b^	8.32 ± 0.56^b^
BC21	Chickpea	10	0	10.44 ± 0.53^d^	506 ± 10^d^	1399 ± 27^b^	0.656 ± 0.01^a^	11.66 ± 1.44^c^
BC22	Chickpea	10	50	7.76 ± 0.59^b^	394 ± 17^a^	1399 ± 27^b^	0.718 ± 0.02^b^	17.80 ± 2.32^e^
BC31	Chickpea	30	0	9.28 ± 0.48^d^	449 ± 18^b^	1399 ± 27^b^	0.693 ± 0.01^b^	10.74 ± 2.07^c^
BC32	Chickpea	30	50	6.08 ± 0.18^a^	344 ± 16^a^	1399 ± 27^b^	0.760 ± 0.01^c^	17.01 ± 1.80^e^
BL21	Lentil	10	0	8.03 ± 1.03^c^	515 ± 42^d^	1394 ± 35^c^	0.647 ± 0.01^a^	8.70 ± 1.15^b^
BL22	Lentil	10	50	10.3 ± 0.36^d^	484 ± 28^c^	1394 ± 35^c^	0.652 ± 0.02^a^	9.64 ± 0.60^c^
BL31	Lentil	30	0	9.94 ± 0.68^d^	410 ± 13^b^	1394 ± 35^c^	0.710 ± 0.01^b^	7.54 ± 0.47^a^
BL32	Lentil	30	50	7.04 ± 0.18^b^	386 ± 31^a^	1394 ± 35^c^	0.718 ± 0.02^b^	13.78 ± 1.50^d^
BM42	Lentil-chickpea	15	50	7.37 ± 0.51^b^	369 ± 15^a^	1405 ± 50^d^	0.734 ± 0.01^b^	12.82 ± 0.41^d^

Samples with different letters in the same column differ significantly (*p* < 0.05).

## Data Availability

The data used to support the findings of this study will be available from the corresponding author upon request.
